# ISO-66, a novel inhibitor of macrophage migration inhibitory factor, shows efficacy in melanoma and colon cancer models

**DOI:** 10.3892/ijo.2014.2551

**Published:** 2014-07-22

**Authors:** KYRIAKI IOANOU, KAI FAN CHENG, GREGG V. CRICH LOW, ANASTASIOS I. BIRMPILIS, ELIAS J. LO LIS, OURANIA E. TSITSILONIS, YOUSEF AL-ABED

**Affiliations:** 1 Department of Animal and Human Physiology, Faculty of Biology, University of Athens, Athens 15784, Greece; 2 Center for Molecular Innovation, The Feinstein Institute for Medical Research, Manhasset, NY 11030, USA; 3 Department of Pharmacology, Yale University, New Haven, CT 06510, USA

**Keywords:** MIF, MIF inhibitor, ISO-66, cancer, melanoma, colon

## Abstract

Macrophage migration inhibitory factor (MIF) is a pleiotropic pro-inflammatory cytokine, which possesses a contributing role in cancer progression and metastasis and, thus, is now considered a promising anticancer drug target. Many MIF-inactivating strategies have proven successful in delaying cancer growth. Here, we report on the synthesis of ISO-66, a novel, highly stable, small-molecule MIF inhibitor, an analog of ISO-1 with improved characteristics. The MIF:ISO-66 co-crystal structure demonstrated that ISO-66 ligates the tautomerase active site of MIF, which has previously been shown to play an important role in its biological functions. *In vitro*, ISO-66 enhanced specific and non-specific anticancer immune responses, whereas prolonged administration of ISO-66 in mice with established syngeneic melanoma or colon cancer was non-toxic and resulted in a significant decrease in tumor burden. Subsequent *ex vivo* analysis of mouse splenocytes revealed that the observed decrease in tumor growth rates was likely mediated by the selective *in vivo* expansion of antitumor-reactive effector cells induced by ISO-66. Compared to other MIF-inactivating strategies employed *in vivo*, the anticancer activity of ISO-66 is demonstrated to be of equal or better efficacy. Our findings suggest that targeting MIF, via highly specific and stable compounds, such as ISO-66, may be effective for cancer treatment and stimulation of anticancer immune responses.

## Introduction

Macrophage migration inhibitory factor (MIF), a cytokine first identified in 1966, is expressed fairly ubiquitously and has both extracellular and intracellular roles (reviewed in ref. 1). MIF has been implicated in auto-immune and infectious disease, and cancer (reviewed in ref. 1). The causative role of MIF in cancer progression was initially linked to its increased expression by a variety of cancer cells, including prostate, colon, hepatocellular, lung, ovarian, in addition to melanoma, glioblastoma and neuroblastoma (2). In these cancers, MIF overexpression has been associated with a concomitant increase in: a) tumor invasion/migration, b) metastasis and c) angiogenesis. More recent data have additionally identified the role of the host MIF in regulating tumor growth. In support of the latter, the role of MIF in regulating tumor angiogenesis in the B16–F10 melanoma model has recently been investigated (3). A combination either of B16–F10 or of shRNA targeting of MIF in the same melanoma cells with a MIF knockout (−/−) genetic background, resulted in significant reduction of tumor growth (by 47%), when compared to wild-type mice. Furthermore, reduced growth of CT26 colon tumors (by 75%) in MIF^−/−^ mice was reported (4). Finally, host macrophage-produced MIF was shown to polarize tumor-associated macrophages (TAMs) towards an immunosuppressive M2 phenotype, whereas MIF inhibition or gene loss (MIF^−/−^) reversed TAM functionalities to M1-type (5). These studies, in conjunction with the previous reports, establish MIF as a promising anticancer drug target.

An increase of MIF levels positively correlates with a poor prognosis in cancer (6–8). Anti-MIF neutralizing antibodies (Abs), MIF-directed siRNA/shRNA or antisense oligonucleotides and compounds hindering MIF secretion have been tested *in vitro* and in preclinical models with notable results (9,10). A more attractive approach to decrease MIF upregulation is the utilization of small molecule MIF inhibitors, which advantageously block the activity of both cancer cell- and host cell-secreted MIF. ISO-1, the ‘gold standard’ inhibitor of MIF, was designed to selectively ligate the tautomerase catalytic site of MIF which is known to neutralize its pro-inflammatory activity (1,11,12). *In vitro* MIF reduction by ISO-1, has been reported to effectively reduce cancer cell proliferation, migration, and invasion of the human lung adenocarcinoma A549 (10,13) decrease the proliferation and invasiveness of prostate cancer DU-145 cells (9), restore contact inhibition of proliferation of LN 229 and LN -18 glioblastoma cells (14), reduce cell migration and invasion of HS683 glioma cells (15), and suppress the proliferation of the murine colorectal cancer cells CT-26 (16). Previous studies have also addressed the role of ISO-1 in prostate and colorectal cancer *in vivo* (9,16). In both mouse models, ISO-1 treatment resulted in significant reduction of the tumor volume or weight, despite the lack of an optimal dosing regimen.

In our search for more effective MIF inhibitors, we herein identify ISO-66, as a potent inhibitor of MIF. We show that ISO-66 enhances the cytotoxicity of human lymphocytes *in vitro* and when administered to mice with established tumors (melanoma and of the colon), is effective in suppressing tumor growth *in vivo*. We believe ISO-66 offers promise as a MIF-reducing therapy in cancer and other MIF-implicated diseases.

## Materials and methods

### Preparation of ISO-66

All solvents were HPL C-grade from Fisher Scientific. Silica gel (Selecto Scientific, 32–63 μm average particle size) was used for flash column chromatography (FCC). Aluminum-backed Silica Gel 60 with a 254-nm fluorescent indicator TLC plates were used. Spots on TLC plates were visualized under a short wavelength UV lamp or stained with I_2_ vapor. NMR spectra were collected on a Jeol Eclipse 400 spectrometer at 400 MHz for ^1^H NMR spectra and 100 MHz for the ^13^C NMR spectra. Coupling constants are reported in Hertz (Hz), and chemical shifts are reported in parts per million (ppm) relative to deuterated solvent peak. The coupling constants (*J*) are measured in Hertz (Hz) and assigned as s (singlet), d (doublet), t (triplet), m (multiplet) and br (broad). High-resolution mass spectra were carried out at the Mass Spectrometry Facility at the Hunter College of the City University of New York.

To a solution of 3-fluoro-4-hydroxybenzaldoxime (1 g; 6.45 mmol) in anhydrous DMF (120 ml) was added NCS (1.03 g; 7.74 mmol). The reaction mixture was stirred for 5 h at room temperature affording the chloro oxime. To this solution, 4-penten-2-one (1.0 ml; 9.7 mmol) (oxidize 4-penten-2-ol with PCC in DCM) was added, followed by the dropwise addition of triethylamine (1.34 ml; 9.68 mmol) in DMF (12 ml). The reaction mixture was stirred under N_2_ at room temperature for 48 h. The solvent was removed *in vacuo* and the residue was taken up in EtOAc. The EtOAc solution was washed with 0.5 NHCl, water, brine, and dried with anhydrous MgSO_4_. The filtrate was evaporated to dryness and the residue was purified by FCC (hexane:EtOAc 4:3) to yield ISO-66 as a pale yellow solid (0.6 g; 39%). ^1^H NMR (500 MHz, Methanol-d_4_) δ 7.40 (d, 1H), 7.30 (d, 1H), 6.96 (m, 1H), 5.05 (m, 1H), 3.53 (m, 1H), 3.03 (m, 2H), 2.86 (m, 1H), 2.21 (s, 3H); ^13^C NMR (125 MHz, Methanol-d_4_) δ 31.43, 42.08, 79.24, 116.07, 116.23, 119.85, 123.54, 125.70, 149.46, 152.76, 154.68, 158.84, 209.48; HR-MS(ES) *m/z* (M+H) 238.0871 (found); 238.0873 (calculated). Compound KF III 53Y, the prodrug of ISO-66, was prepared from the ISO-acid chloride with bis-trimethylsilyl malonate via methods reported in the literature (17,18). KF III 53Y has better solubility than ISO-66, but undergoes fast decarboxlyation to form ISO-66 upon formulation in buffer.

### MIF tautomerase inhibition assay

MIF tautomerase activity was measured using a UV-Visible spectrophotometer (Shimadzu, UV1600U). A fresh stock solution of *L*-dopachrome methyl ester was prepared at 2.4 mM through oxidation of *L*-3,4-dihydroxyphenylalanine methyl ester with sodium periodate. One μl of MIF solution (800–900 μg/ml) and 1 μl of a dimethyl sulfoxide (DMSO) solution with various concentrations of the MIF inhibitor were added into a plastic cuvette (10 mm, 1.5 ml) containing 0.7 ml assay buffer (50 mM potassium phosphate, pH 7.2). Then, activated *L*-dopachrome methyl ester solution (0.3 ml) was added to the assay buffer mixture. Activity was determined at room temperature and spectrometric measurements were made every 5 sec at λ = 475 nm for total 20 sec, by monitoring the rate of decolorization of *L*-dopachrome in comparison to a standard solution.

### Crystallization and X-ray data collection

Recombinant human MIF was expressed in *E. coli* and purified as described (19). Briefly, cells were lysed using a French Pressure Cell, the lysate was clarified by centrifugation, and the supernatant liquid was filtered with a 0.22-μm filter. The filtered supernatant was purified by ion-exchange in 20 mM Tris (pH 7.5), 20 mM NaCl using a DEAE and an SP column connected in series. MIF is found in the flow-through. Flow-through fractions were collected, and fractions containing pure MIF were pooled and concentrated.

A stock solution of 1.2 mM MIF in 20 mM Tris (pH 7.5), 20 mM NaCl, and a stock of 0.10 M KF III 53Y (the carboxylated derivative/prodrug, of ISO-66) in DMSO were used to prepare a solution of 1.1 mM MIF, 10 mM KF III 53Y, 18 mM Tris (pH 7.5), 18 mM NaCl, 10% DMSO. The KF III 53Y spontaneously decarboxylated non-enzymatically, forming ISO-66. Crystallization was performed using the hanging-drop vapor diffusion method. Two μl of the MIF-ISO-66 complex was mixed with an equal volume of reservoir comprised of 2 M (NH_4_)_2_SO_4_, 0.1 M Tris (pH 7.5), 3% isopropanol. Crystals grew in three to five weeks. X-ray diffraction data were collected from a single crystal at station X29 of the National Synchrotron Light Source at Brookhaven National Laboratory. Crystal dimensions were ~0.40×0.25×0.23 mm. Immediately prior to mounting the crystal, it was soaked briefly in cryoprotective solution containing 2.25 M NaCl, 2 M (NH_4_)_2_SO_4_, 50 mM Tris (pH 7.5). An ω-sweep of 100° of data were collected with 1.0809 Å radiation. Data were processed using HKL2000. Data collection statistics are presented in Table I.

### Structure determination

The program AMoRe was used for molecular replacement. Protein coordinates from the complex of MIF with OXIM-11 (20) was used as the search model. Refinement was performed primarily using the program CNS (21). Refmac5 was also used for refinement. Occupancy refinement was performed using CNS. Refined occupancies were normalized so that the sum of the occupancies of corresponding atoms was 1.0. A TLS model was employed in the Refmac5 refinement. The initial TLS model was found using the TLSMD web server. Manual manipulation of the model was performed using O and COOT. The structure was deposited into the RCSB Protein Data Bank. Crystallographic statistics are presented in Table I.

### Cell lines and culture conditions

The human FM3 (melanoma), HCT-116 (colorectal carcinoma), K562 (leukemia), Daudi (Burkitt’s lymphoma), and the murine B16-F1 (melanoma), CT-26.WT (colorectal carcinoma), YAC-1 (lymphoma), WE HI 164 (fibrosarcoma) cell lines, peripheral blood mononuclear cells (PBMCs) and their subpopulations and mouse spleen cells were cultured in RP MI-1640, supplemented with 10% heat-inactivated fetal bovine serum (FBS), 2 mM L-glutamine, 10 mM Hepes, 50 μM β-mercaptoethanol, 5 μg/ml gentamycin, 10 U/ml penicillin and 10 U/ml streptomycin (all from Lonza, Cologne, Germany) (thereafter referred to as complete medium), at 37°C, in a humidified 5% CO_2_ incubator. ISO-66 was dissolved in DMSO at 50 mM, aliquoted and stored at −20°C. Serial dilutions of stock solutions were freshly prepared prior to their use.

### Cell isolation and separation

Buffy coats were collected from 7 healthy blood-donors. Prior to blood withdrawal, individuals gave their informed consent according to the regulations approved by the 2nd Peripheral Blood Transfusion Unit and Haemorophilic Centre, ‘Laikon’ General Hospital Institutional Review Board, Athens, Greece.

PBMCs were isolated by centrifugation over Ficoll-Histopaque (Lonza) density gradient, resuspended in complete medium or cryopreserved in FBS-10% DMSO for later use. Purified natural killer (NK; CD56^+^) cells were obtained using an immunomagnetic isolation procedure. Briefly, 5–10×10^6^ PBMCs were incubated for 15 min at 4°C with 20 μl of anti-CD56 monoclonal Ab conjugated to magnetic beads (Miltenyi Biotec, Auburn, CA, USA). CD56^+^ cells were isolated by positive selection on an MS column (Miltenyi), according to the manufacturer’s instructions. Purity of the isolated populations was tested by flow cytometry (FACSCalibur, Becton-Dickinson, Mountain View, CA, USA), using FITC- and PE -conjugated anti-CD3 and anti-CD56 monoclonal Abs (BD Pharmingen, San Diego, CA, USA). In all cases, purity of the NK population was >90%.

For lymphokine-activated killer (LAK) cell generation, PBMCs were seeded in 24-well plates (1×10^6^ cells/ml; 2 ml/well) and cultured for 5 days in complete medium supplemented with 1,000 IU/ml recombinant human interleukin (IL)-2 (Proleukin, Roche, CA, USA). Culture medium containing IL-2 was renewed every other day. On day 5, LAK cells were harvested and tested as described.

Monocytes were isolated by seeding PBMCs in 6-well plates (5×10^6^/ml; 3 ml/well) and let adhere for 2 h at 37°C. The non-adherent cell fraction (comprising >80% CD3^+^ cells as assessed by flow cytometry) was collected and cryopreserved for later use in T cell stimulation cultures.

### Proliferation assay

FM3, HCT-116, B16-F1 and CT-26.WT cancer cells were seeded into 96-well U-bottom microplates (Costar, Cambridge, MA, USA; 20–25×10^3^ cells/ml; 200 μl/well) and pre-incubated for 24 h to adhere. ISO-66 was serially diluted at various concentrations (1 mM-0.04 μM), added to the wells and incubated for 72 h. All cultures were set in triplicates, whereas cultures set in complete medium or containing an equivalent amount of DMSO (2% v/v) or doxorubicin (0.5 μM; Sigma-Aldrich, Japan) were used as controls. For the last 18 h, ^3^[H]-thymidine (Amersham Pharmacia Biotech, Amersham, Bucks, UK) was added and inhibition of proliferation was determined as described (22).

### Stimulation of PBMCs, NK, LAK and T cells with ISO-66

PBMCs were seeded in 48-well plates (1×10^6^ cells/ml; 1 ml/well), ISO-66 was added at final concentrations of 1 mM-1 μM and cells were cultured for 3 days. PBMCs cultured in plain complete medium served as controls. NK cells were isolated by magnetic beads and used as effector cells (E) versus K562 (NK-sensitive) targets (T), in standard ^51^Cr release assays at an E:T ratio of 10:1. Similarly cultured LAK cells were also used as effectors versus K562 and Daudi (LAK-sensitive) targets at an E:T ratio of 50:1.

T cells were stimulated with a pool of tumor antigenic peptides, extracted from the cell surface of FM3 and HCT-116 [acid wash extract, AWE (25)], as previously described (23). In brief, on day 0 monocytes were irradiated at 30 Gy, washed and co-incubated with autologous T cells, at a monocyte:lymphocyte ratio of 1:5. AWE extracted from 20×10^6^ FM3 or HCT-116 cells and ISO-66 at a final concentration of 10 μM, were added to each well. Cultures set without ISO-66 served as controls. On day 5, lymphocytes were restimulated with autologous irradiated monocytes and FM3- or HCT-116-AWE. On days 2, 5, 7 and 9, IL-2 (40 IU/ml) and ISO-66 (10 μM) were added to the culture medium. T cells were harvested on day 11 and tested for their cytotoxic activity versus FM3, HCT-116 and Daudi targets, using standard ^51^Cr-release assays, at an E:T ratio of 50:1.

### In vivo melanoma and colon cancer mouse models

Female mice, 6–8 weeks of age, 15–20 g of weight, of the strains C57BL/6 and Balb/C were purchased from Harlan Laboratories (Udine, Italy). All mice were maintained under conditions and protocols in accordance with Law 2015/27/2.1992, Presidential Decree 160/3.5.1991 and the Directive 86/609/EE C/24.11.1986 of the Council of Europe on Animal Welfare. The study was approved by the Ethics and Biosafety Committee, Subcommittee on Ethics, Department of Biology, University of Athens and all experiments were conducted following the guidelines of the aforementioned committee.

B16-F1 (syngeneic to C57BL/6 mice) and CT-26.WT (syngeneic to Balb/C mice) were expanded to sufficient numbers in complete medium. On day 0, C57BL/6 and Balb/C mice were subcutaneously (s.c.) inoculated with 1×10^5^ B16-F1 or 1×10^6^ CT-26.WT (in 250 μl PBS), respectively, and mice of each strain were randomly assigned to 5 groups (8 mice/group). On days 12–14, when tumors were palpable, and for 20 consecutive days, mice received intraperitoneally (i.p.) PBS or DMSO (control groups), or ISO-66 (all diluted in 300 μl PBS) once daily. ISO-66 was administered at 180 μg/dose/animal. DMSO was administered at a final concentration of 4% v/v, equivalent to the amount present in ISO-66 injections. Tumor growth rate was recorded every 2–3 days by measuring the major and minor axes of the tumors formed with a digital caliper. Measurements were transformed into tumor volume using the formula: tumor volume (cm^3^) = major axis × minor axis^2^ × 0.5. On days 34 (for C57BL/6) and 40 (for Balb/C), when mean tumor volumes of the control groups exceeded 1.5–2 cm^3^, animals were euthanized and spleens were aseptically excised from 3 mice/group. Splenocytes were isolated from individually homogenized spleens and immediately tested for their cytotoxicity versus B16-F1, CT-26.WT, YAC-1 and WE HI-164 targets in standard ^51^Cr-release assays.

### Cytotoxicity assay

Target cells (T) were ^51^Cr-labelled according to Skopeliti *et al* (24) and co-cultured with the effectors (E) at the indicated E:T ratios. After 18 h at 37°C, 5% CO_2_, 100 μl of each supernatant was removed and isotope was measured in a γ-counter (1275 Minigamma, LKB Wallac, Turku, Finland). Targets were incubated with 3 NHCl and in complete medium to determine maximal and spontaneous isotope release, respectively. All cultures were set in triplicates. Percentage of specific cytotoxicity was calculated according to the formula: (cpm experimental - cpm spontaneous)/(cpm maximal - cpm spontaneous) × 100.

### Statistical analysis

Data were analyzed by the Student’s t-test and statistical significance was presumed at significance level of 5% (p<0.05). Tumor size among groups was compared with Wilcoxon’s signed rank test.

## Results

### ISO-66 as an inhibitor of MIF

**Figure 1:**
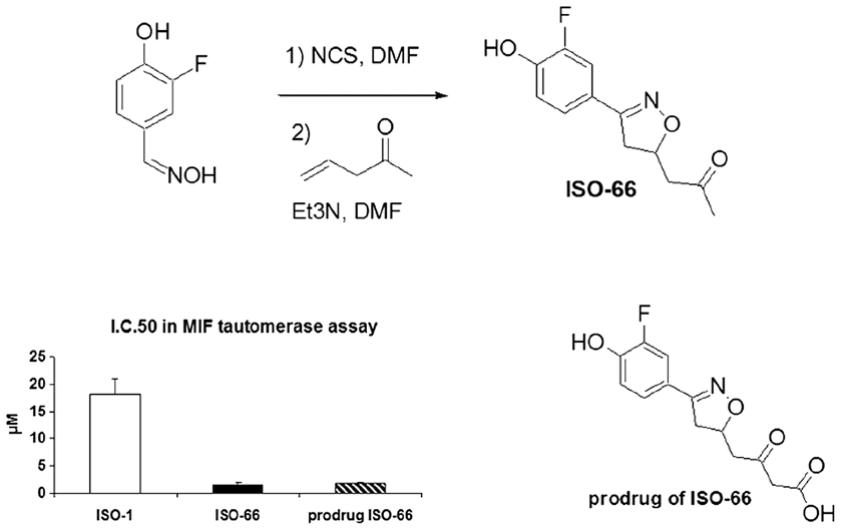
Synthesis of ISO-66 and IC_50_ in MIF tautomerase assay of ISO-1, ISO-66 and the prodrug of ISO-66 (ISO-1, 18.2±2.8 μM; ISO-66, 1.5±0.4 μM; prodrug of ISO-66, 1.8±0.1 μM).

We have identified ISO-66, a novel analog of ISO-1, as a potent inhibitor of MIF tautomerase activity (Fig. 1). The rationale for the design of ISO-66 is based on our previous experience with ISO-1 design and structure activity relationship (SAR) studies. For instance, the ester functional group in ISO-1 has been shown to play an important role in binding to MIF, as evident by SAR studies and MIF: ISO-1 co-crystal structure (11). However, the methyl ester may hydrolyze and this could limit future application of ISO-1. We hypothesized that the methyl ester could be replaced by a ketone group and came up with the structure of ISO-66. ISO-66 was synthesized in three steps and characterized by NMR and mass spectrometry. ISO-66 was found to inhibit MIF tautomerase activity with an IC_50_ of 1.5±0.4 μM compared to 18.2±.8 μM for ISO-1. It is superior to ISO-1 in both its potency and stability profile. To improve the solubility of ISO-66, the β-keto carboxylate was also synthesized (referred to as KF III 53Y/prodrug of ISO-66); however, it was found by mass spectrometry that KF III 53Y spontaneously decarboxylates to form ISO-66. This finding was also supported by structural studies, in which MIF was co-crystallized with KF III 53Y.

### Structure of MIF-bound ISO-66

**Figure 2:**
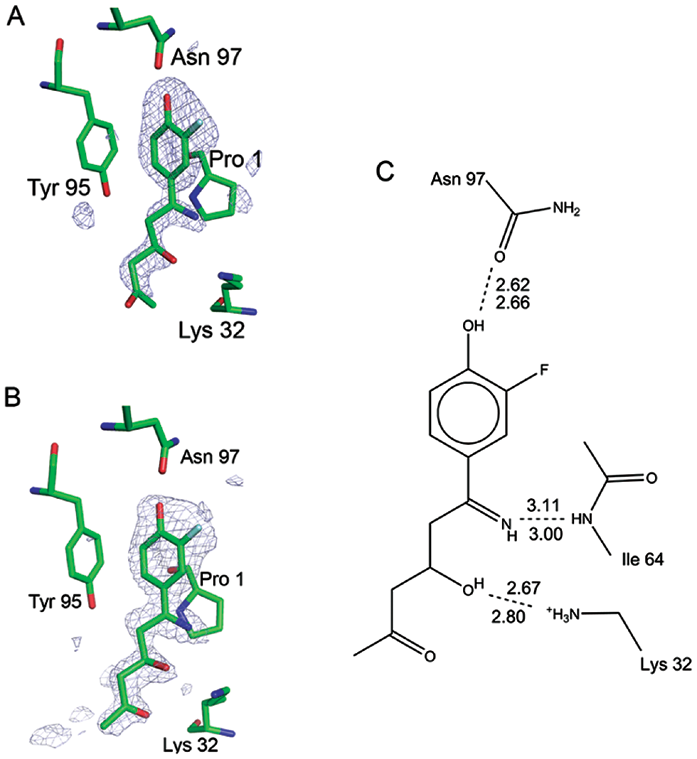
Crystallographic structure of modified ISO-66 bound to MIF. (A and B) The F_o_-F_c_ electron density, calculated with the omission of all inhibitor atoms, contoured at 2.5σ. (A) The fit of the inhibitor in the solvent-exposed active site (active site C). (B) The fit in active site B, which has better-defined density, presumably due to crystal contacts. (C) Schematic representation of the hydrogen bonding interactions of modified KF III 53Y/prodrug of ISO-66 with MIF. Numbers listed above are distances in Ångstroms from active site C, and the lower ones are those from active site B.

ISO-66 was found bound in the MIF tautomerase active sites, with the *meta*-fluoro-*para*-hydroxyphenyl ring buried in the innermost part of the cleft, as expected. What was unexpected, however, was that the five-membered ring of the inhibitor had been opened. The bond between the nitrogen and oxygen was cleaved, resulting in free imine and hydroxyl groups (Fig. 2). An open ring structure was seen in all three active sites. Fig. 2 shows the electron density for the inhibitor in active site C, in which no active site residues participate in crystal contacts, clearly revealing the absence of the five-membered ring. The electron density is also shown for active site B, in which the density is better defined due to the crystal contacts. The presence of the modified (ring-opened) inhibitor in the active site demonstrates that it is itself a potential inhibitor of MIF’s physiological activity.

Both non-polar and polar interactions occur between the modified inhibitor and MIF. The *m*-fluoro-*p*-hydroxyphenyl ring is buried in the hydrophobic cavity of the protein. The hydroxyl group shares a hydrogen bond with Asn 97 at the base of the cavity. The fluorine atom has Van der Waals contacts with the side chain of Met 101 and the β-methylene group of His 62. Electron density for the fluorine atom is only found on one side of the ring (Fig. 2), demonstrating that the ring does not stochastically flip between the two states 180° apart. Rotating the ring by 180° would cause steric conflict between the Tyr 95 and the electronegative fluorine, which would point toward the Tyr 95 aromatic π-orbital if the inhibitor’s aromatic ring was in such an orientation. Lys 32 donates a strong hydrogen bond to the hydroxyl generated by the ring-opening reaction. The nitrogen that was formerly of the five-membered ring accepts a hydrogen bond from the main chain amide of Ile 64.

**Figure 3:**
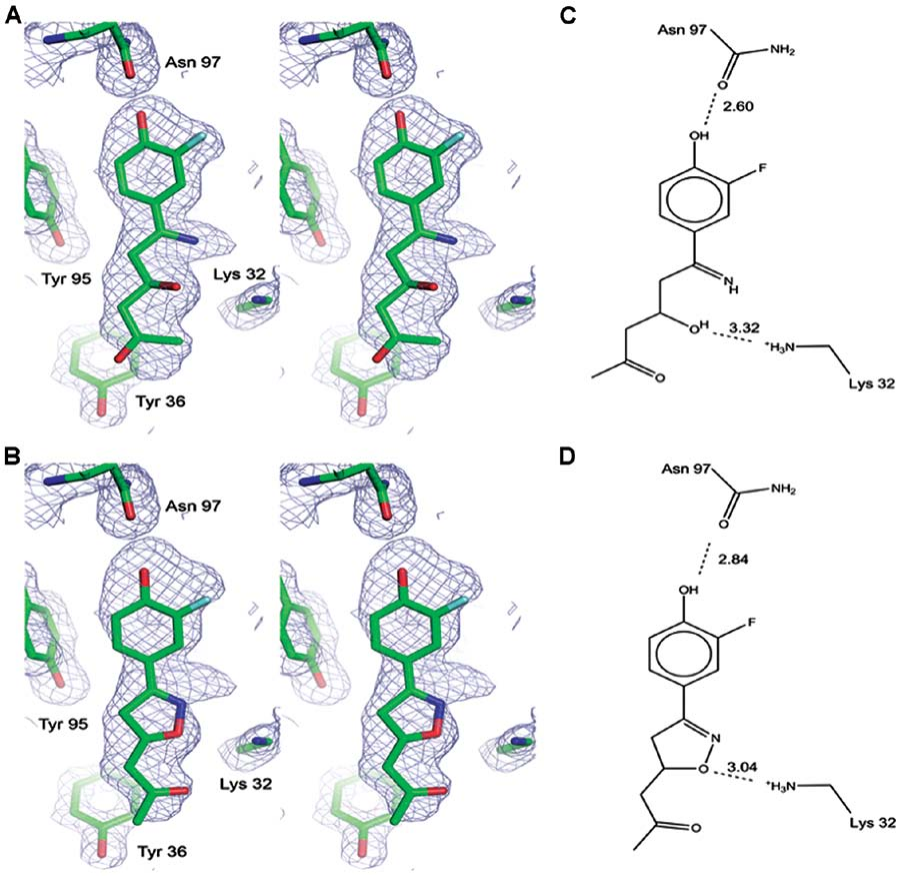
Comparison of the binding of MIF with intact ISO-66 to that with the modified inhibitor. (A) Stereo view of the modified KF III 53Y/prodrug of ISO-66, as bound in active site A. Shown is 2F_o_-F_c_ electron density, omitting only the intact KF III 53Y/prodrug of ISO-66, contoured at 1σ. Note that, while most of the inhibitor fits the electron density, the methyl ketone group does not fit properly. (B) The poor fit in (A) is explained by the presence of a partial occupancy inhibitor with an intact five-membered ring. The electron density map is the same as shown in (A). (C and D) Comparison of the hydrogen bonding interactions between the forms with the five-membered ring open (C) and closed (D) in active site A. Distances are in Ångstroms.

In active site A, however, there was excess electron density around the methylene and terminal methyl ketone group of the inhibitor (Fig. 3A). It is very interesting that this excess density can be explained by the intact, ring-closed form of the inhibitor, having partial occupancy (Fig. 3B). Both the ring-opened and ring-closed forms were modeled into the electron density, having occupancies of 0.88 and 0.12, respectively. The low occupancy of the intact inhibitor has no or little effect on the electron density in the phenyl portion where the B-factors for the modified inhibitor are lower than those of the intact form. However, the higher B-factors on the methyl ketone side of the modified inhibitor yields deterioration of the electron density there, where we instead see the intact form. The intact inhibitor also accounts for the extra breadth of electron density located at the five-membered ring, as shown in Fig. 3.

Both the intact and modified forms of ISO-66 interact with MIF in active site A. The interactions of the modified compound with MIF in active site A are similar to those in the other two active sites. The hydrogen bond between the nitrogen of the ring-opened inhibitor and the Ile 64 backbone amide is stretched (3.46 Å) relative to the other two active sites. The binding interactions of intact ISO-66 with MIF are as follows: Lys 32 donates a hydrogen bond to the oxygen atom of the five-membered ring and is also within hydrogen bonding distance to the ring nitrogen. The phenyl ring is positioned in the MIF hydrophobic pocket, but does not fit as deep into the cavity as the phenyl ring of the modified inhibitor. The hydroxyl group of the phenyl ring of the intact inhibitor ISO-66 shares a hydrogen bond with Asn 97. However, this is not as strong as the hydrogen bond between Asn 97 and the modified inhibitor (Fig. 3). This presents the structural view of how ISO-66 binds to its target MIF, providing a means for it to have the antitumor effects described in this report. Furthermore, this suggests the open-ring form we discovered through the crystallographic analysis could also be a potential chemotherapeutic lead compound, which may be investigated in future studies.

### ISO-66 does not affect cancer cell proliferation in vitro

**Figure 4:**
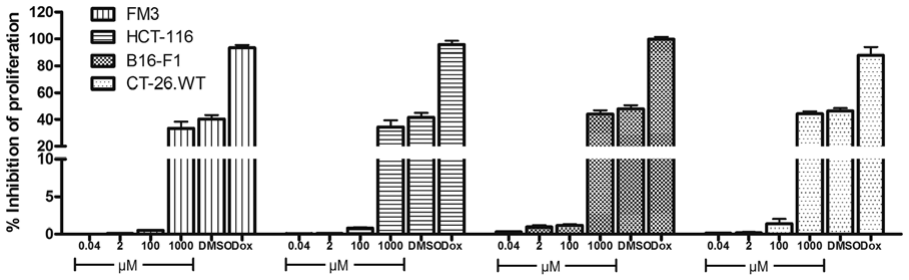
ISO-66 does not inhibit cancer cell proliferation. FM3, HCT-116, B16-F1 and CT-26.WT cells were incubated with various concentrations of ISO-66 (1 mM-0.04 μM), DMSO (2% v/v) and doxorubicin (Dox; 0.5 μM) for 72 h. The percentage of DMSO corresponds to the equivalent amount used to dilute 1 mM of ISO-66.

To assess whether ISO-66 is directly cytotoxic to cancer cells, we tested its effect in inhibiting the proliferation of the human and mouse melanoma (FM3 and B16-F1) and colon cancer cells (HCT-116 and CT-26.WT). In all experiments, the equivalent amount of DMSO (2% v/v) present in the highest ISO-66 concentration tested (1 mM), and the chemotherapeutic drug doxorubicin (0.5 μM) were used as controls. Our results showed that low concentrations of ISO-66 did not affect the proliferation of cancer cells, whereas at the highest ISO-66 concentration tested, an analogous inhibition of proliferation (~50%) was also caused by the equivalent amount of DMSO (Fig. 4).

### ISO-66 enhances NK and LAK cell cytotoxicity

**Figure 5:**
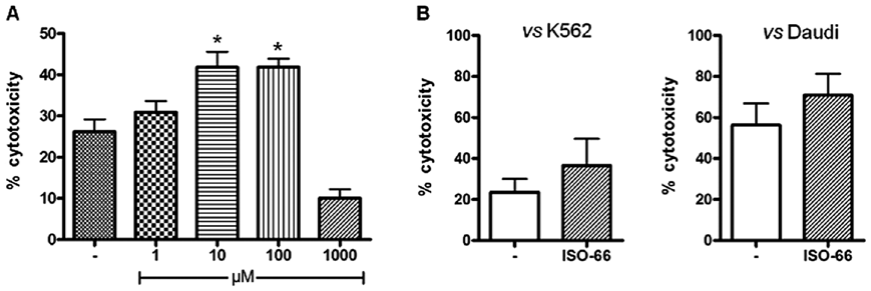
ISO-66 enhances NK and LAK cell cytotoxicity. (A) PBMCs were incubated for 3 days with ISO-66 (1 mM-1 μM), NK cells were purified and tested as effectors against K562, at an E:T ratio of 10:1. ^*^p<0.05 compared to (−). (B) LAK cells were incubated for 3 days with ISO-66 (10 μM) and tested as effectors against K562 (left) and Daudi (right). The E:T ratio was 50:1. Data are mean cytotoxicities ± SD from 2–5 healthy donors. (−), cells incubated in plain medium.

For assessing the ability of ISO-66 to induce cytolytic PBMC responses *in vitro*, we incubated normal donor-derived PBMCs with 1 mM-1 μM ISO-66 for 3 days and then we isolated the CD56^+^ cells from the cultures. Our titration experiment (Fig. 5A) showed that NK cell cytotoxicity versus K562 cell targets was equally enhanced by 16% upon incubation with 10 or 100 μM ISO-66. We further generated LAK cells from PBMCs of healthy donors and cultured them with ISO-66 for 3 days. In the cytotoxicity test, we observed that ISO-66 increased LAK cell cytotoxicity against K562 (36.5% for ISO-66, compared to 23.5% of the control; Fig. 5B), which was further enhanced when Daudi cells were used as targets (70.7% for ISO-66, compared to 56.2% of the control; Fig. 5B). However, for both targets, this increase in LAK cell cytotoxicity did not reach statistical significance.

### ISO-66 enhances T cell cytotoxicity

**Figure 6:**
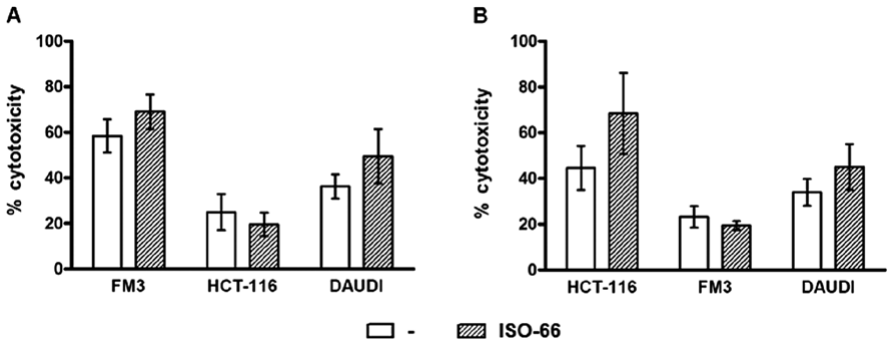
ISO-66 enhances antigen-specific T cell cytotoxicity. T cells were twice stimulated with irradiated, FM3- (A) and HCT-116- (B) AWE -loaded autologous monocytes, in the presence or absence of ISO-66 (10 μM). Recovered T cells were tested as effectors against FM3, HCT-116 and Daudi. The E:T ratio was 50:1. Data are mean percentage cytotoxicity ± SD from 5 healthy donors. (−), cells incubated in plain medium.

We next sought to investigate whether ISO-66 is able to enhance T cell cytotoxicity. As specific antigen source for T cell stimulation, we used a pool of tumor antigenic peptides, detached from MHC molecules present on the surface of melanoma (FM3) and colon cancer (HCT-116) cells. In the presence of autologous irradiated monocytes as antigen-presenting cells, this extract (AWE ) has been already used by us (23) and others (25) to generate *in vitro* tumor-reactive T cell lines. On day 5, T cells were restimulated with autologous AWE -loaded monocytes. To expand AWE -reactive T cells, low dose IL-2 (40 IU/ml) was used. ISO-66 was added to the cultures every other day, while cultures in plain medium served as controls. On day 11, AWE -reactive T cells cultured in the presence of ISO-66 were able to recognize and lyse more efficiently the targets used for AWE preparation (Fig. 6). Specifically, FM3-AWE -stimulated T cells killed FM3 targets (58.4%) and this cytotoxicity was enhanced in the presence of the MIF inhibitor (69.0%). The same T cells marginally lysed allogeneic HCT-116 colon cancer targets (<25%), but showed some LAK cytotoxicity (36.1% against Daudi cells), which was higher when ISO-66 was added in the cultures (49.5%; Fig. 6A). Accordingly, HCT-116-AWE -stimulated T cells killed HCT-116 targets (44.6%) and ISO-66 increased this percentage to 68.5%. HCT-116-AWE -stimulated T cells did not lyse FM3 targets (<25%) and similarly to FM3-AWE -stimulated T cells, they exhibited increased LAK activity as detected by their cytotoxicity against Daudi (45.0% for ISO-66, compared to 33.9% of the control; Fig. 6B).

Although, the enhancement of cytotoxicity induced by ISO-66 was in all cases not statistically significant compared to unstimulated cells, we cannot rule out the possibility that additional T cell stimulations could have resulted in a more pronounced effect. Taken as a whole, our results show that ISO-66 increases the specific and non-specific cytotoxic responses of activated human T cells *in vitro*.

### ISO-66 retards melanoma and colon tumor growth in vivo

To test the anticancer activity of ISO-66 *in vivo*, mice were implanted with syngeneic melanoma and colon cancer cells. We specifically selected these two mouse models in order to be able to compare our results on ISO-66 with already reported data, in which MIF shRN A and ISO-1 were used to block MIF in similar *in vivo* melanoma and colon cancer models, respectively (3,16).

**Figure 7:**
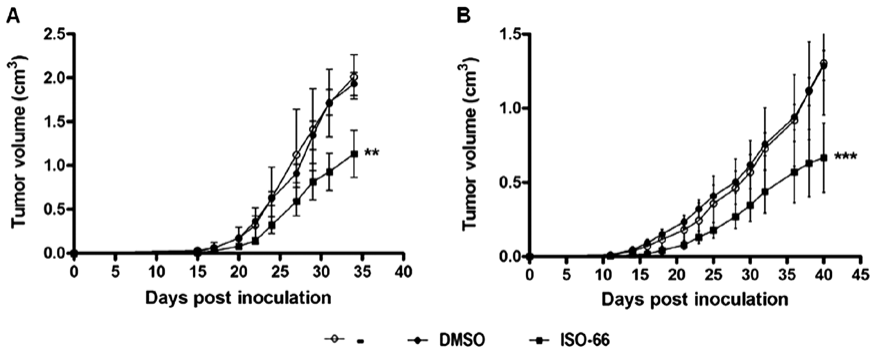
ISO-66 delays melanoma and colon tumor growth. C57BL/6 (A) and Balb/C (B) mice were s.c. inoculated with syngeneic B16-F1 (melanoma) and CT-26.WT (colon cancer) cells, respectively, and i.p. treated with 180 μg/dose/mouse of ISO-66 for 20 consecutive days. Control mice received PBS (−) or DMSO. Tumor growth was monitored for 34 days (A) and 40 days (B). Pooled data from 8 mice/group are shown. ^**^p<0.01; ^***^p<0.001 compared to (−).

Following titration experiments (26), on day 0, C57BL6 and Balb/C mice received s.c. 1×10^5^ B16-F1 or 1×10^6^ CT-26. WT cells (melanoma and colon carcinoma syngeneic cells, respectively). By days 12–14, all mice had developed palpable tumors and were further administered i.p. PBS, DMSO or ISO-66 for 20 consecutive days. The dose of 180 μg ISO-66/animal/day (3.6 mg/mouse) was based on previous reports on the anticancer activity of ISO-1, where, although at different time intervals, 3.2 mg (16) and 4 mg (9) per animal were i.p. administered to tumor-bearing mice, without reported toxicity. In contrast to these studies, in our protocols, we administered ISO-66 daily and for 20 consecutive days, to significantly, if not completely, inactivate MIF constantly produced both by the host and by melanoma (27) or colon cancer cells (28). Tumor growth was monitored until day 34 (for melanoma) or day 40 (for colon cancer). As shown in Fig. 7A, tumor size (expressed in cm^3^) in melanoma-inoculated mice treated with ISO-66, showed a significantly slower increase as compared to controls (i.e., mice receiving PBS or DMSO). On the 34th day post-tumor cell inoculation, control mice were euthanized for ethical reasons (tumor size ≥2 cm^3^), whereas the average tumor volume recorded for animals treated with ISO-66 was 1.2 cm^3^. This tumor reduction of ~45% was statistically significant (p<0.01, compared to controls).

For the colon cancer model (Fig. 7B), by day 40 post-inoculation, both control groups (i.e., mice receiving PBS or DMSO) exhibited a similar rapid tumor development (≥1.5 cm^3^). The therapeutic administration of the MIF inhibitor reduced tumor growth rates in all animals of the ISO-66-treated group. Specifically, the average tumor volume recorded for ISO-66 was 0.55 cm^3^, and this ca. 60% tumor volume reduction was statistically significant compared to controls (p<0.001).

### The in vivo antitumor responses induced upon treatment with ISO-66 are mediated by T cells

**Figure 8:**
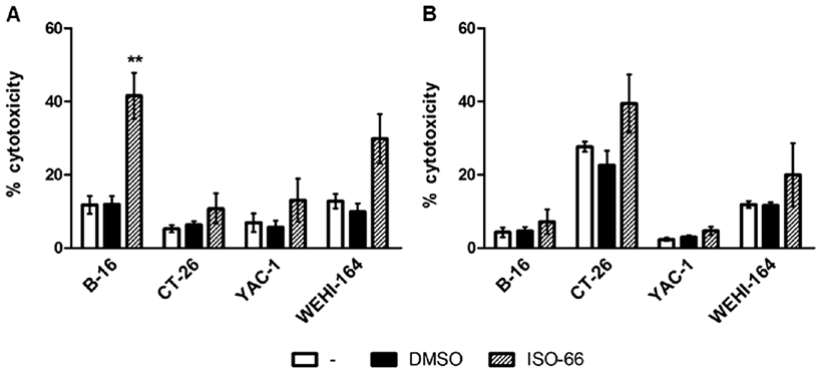
ISO-66 induces tumor-reactive immune responses *in vivo*. Mouse splenocytes were isolated on day 34 for the melanoma (A) and on day 40 for the colon cancer (B) models and used as effectors versus CT-26.WT, B16-F1, YAC-1 (NK-sensitive) and WE HI-164 (LAK-sensitive) target cells, at an E:T ratio of 100:1. Data show mean specific cytotoxicity ± SD from spleen cells of 3 mice per group (see legend of Fig. 7), tested individually. ^**^p<0.01 compared to (−).

To verify whether the *in vivo* reduction of tumor growth was associated with increased antitumor immune responses induced by ISO-66, three mice from each group that developed the smaller tumors were sacrificed on day 34 for melanoma and on day 40 for the colon cancer model. Without additional *ex vivo* stimulation, their splenocytes were used as effectors in ^51^Cr-release assays against the murine NK-sensitive targets YAC-1, the LAK-sensitive WE HI-164, and the syngeneic cells B16-F1 and CT-26.WT. Splenocytes from ISO-66-treated melanoma-inoculated mice were more efficient in killing B16-F1 targets than PBS- or DMSO-treated mice (41.5 versus 11.7 and 11.9%, respectively; p<0.01 compared to PBS; Fig. 8A). The same splenocytes did not lyse the non-syngeneic CT-26.WT colon targets (10.8%), or YAC-1 (13.0%), but showed some cytotoxicity against WE HI-164 (29.8% compared to 12.8% of the control; p<0.05). These results indicate that the MIF inhibitor ISO-66 increased the cytotoxicity of LAK cells and, most importantly, induced the *in vivo* expansion of melanoma-reactive T cells.

Accordingly, spleen cells from mice bearing colon cancer cells when treated with ISO-66, lysed the syngeneic CT-26.WT targets (39.4% compared to 27.6% of the PBS group; Fig. 8B), as well as the LAK-sensitive WE HI-164 (20% compared to 11.9% of the PBS group). Marginal cytotoxicity against the melanoma B16-F1 and YAC-1 was recorded. Thus, from the results obtained from both cancer models, we observe that ISO-66 enhances tumor-reactive (T cell-mediated) and non-specific (LAK cell-mediated) immune responses leading to decreased cancer cell growth.

## Discussion

MIF is a pleiotropic cytokine broadly studied in sepsis and many infectious and auto-immune diseases (1). Recently, a causative role in cancer progression has been attributed to MIF, as it has been reported to inactivate p53 (29), enhance angiogenesis (30), promote metastasis (31) and impair both innate and adaptive immune responses (5,32–34). Therefore, inactivating MIF provides an alternative therapeutic option in anticancer treatment (11). Small-molecule inhibitors of host- and cancer cell-derived MIF have been used to block its activity (35). The most prominent MIF inhibitor, ISO-1, is known to inhibit the proliferation/invasiveness of cancer cell lines *in vitro* (9,10,13–16,36) and tumor growth and vascularization *in vivo* (9,16).

In this study, using the structure of ISO-1 as a scaffold, we designed a novel, more potent and more stable MIF inhibitor. In our initial *in vitro* studies, we observed that ISO-66 did not affect human and mouse cancer cell proliferation, even at concentrations as high as 1 mM. On the contrary, ISO-66 induced the cytotoxic potential of distinct effector cell populations with antitumor activity, namely NK and LAK cells and cytotoxic T cells (CTLs) (37). Specifically, NK cells purified from human PBMCs cultured with ISO-66 showed increased killing of tumor targets (K562) and LAK cells generated with high concentrations of IL-2, when stimulated with ISO-66 efficiently lysed both K562 and Daudi targets. Most importantly, in the presence of ISO-66, tumor antigen-reactive CTLs generated *in vitro*, recognized and effectively killed the relative tumor-antigen expressing targets. Our results are in agreement with previous reports showing that MIF hampers anticancer immunity by inhibiting tumor-specific CTL and NK cell activity (33,34). The mechanisms responsible for this CTL deficiency, probably include MIF-induced downregulation of receptors involved in tumor cell recognition (34) and/or excess T cell activation, leading to cell death (33). MIF is also known to impair the ability of NK cells to release cytolytic perforin granules (32). Additionally, it has been suggested that MIF’s effect is optimally exerted on activated cells, whereas normal functions of resting cells evolve independently of MIF (9). Our results are in agreement with this scenario, as NK, IL-2- activated LAK cells and antigen-stimulated CTLs, increased their cytotoxicity in the presence of ISO-66. Although ISO-1 has been reported to inhibit proinflammatory cytokine and IFN-γ secretion by human PBMCs or subpopulations thereof (38,39), the enhanced functional responses of NK, LAK and CTLs as observed in our *in vitro* system, do not support such an ISO-66-induced suppressive effect. More likely, ISO-66 acts by blocking the activity of MIF produced by host macrophages, possibly also T cells, allowing effectors to exert their lytic functionalities.

Translating our *in vitro* observations *in vivo*, the therapeutic anticancer activity of ISO-66 was tested in C57BL/6 and Balb/C mice inoculated with syngeneic melanoma B16-F1 and colon CT-26.WT tumor cells, respectively. In line with similar doses of ISO-1 given by others (9,16), we observed that injecting a total of 3.6 mg of ISO-66 per mouse was well-tolerated and did not cause any adverse effects. Further, it led to a statistically significant retardation of tumor growth in both models (45% for melanoma and ca. 60% for colon carcinoma, compared to controls). Although the therapeutic protocol of the *in vivo* ISO-66 administration used in our study differs from other regimens used to date in terms of intervals between injections, our results are comparable with previously reported data on the antitumor activity both of ISO-1 and anti-MIF Abs. Specifically, He *et al* (16) recorded a 25% inhibition of CT-26 colon tumors upon treatment with either ISO-1 or anti-MIF Abs administered twice per week, whereas Ogawa *et al* (40) reported a much higher inhibition (55%) of the same tumor upon a more frequent (every other day) treatment with anti-MIF Abs. These data support our rationale of maximizing MIF inactivation by injecting mice with ISO-66 daily for 20 consecutive days. Of greater importance are the recent elegant studies of Girard *et al* (3) and Choi *et al* (4) in which MIF^−/−^ mice inoculated with B16-F10 melanoma and CT26 colon cancer cells, respectively, exhibited a 47% and 75% tumor reduction compared to wild-type animals. Melanoma growth in wild-type mice given daily ISO-66 (Fig. 7A) resembles that observed in MIF^−/−^ mice (3). Similarly, colon tumor growth in our model (Fig. 7B), greatly coincides the growth recorded in MIF^−/−^ mice (4). Therefore, we can propose that repetitive, daily dosing of ISO-66 effectively reduces the cancer-promoting effects of MIF.

This was further confirmed upon analyzing the *ex vivo* cytotoxicity of spleen cells from mice administered ISO-66 against the inoculated tumors. We used splenocytes from 3 animals per group with minimal tumor load, as these most likely had developed the highest percentages of cytotoxic effectors. Our results revealed that splenocytes from mice therapeutically administered ISO-66 exhibited increased specific lysis only of the syngeneic cancer cells, both for melanoma and colon cancer. Although we also observed a non-negligible percentage of non-specific cytotoxic responses enhanced by ISO-66, these accounted for <50% of the syngeneic tumor cell killing. To our knowledge, our results for the first time suggest that successful MIF inactivation *in vivo* by ISO-66 leads to restoration of the impaired tumor-reactive lymphocyte responses in cancer-bearing mice. Due to ethical reasons (Guidelines of Ethics and Biosafety Committee) we could not follow murine tumor growth for a prolonged period, but based on our results we could safely speculate that ISO-66 administration would also lead to prolonged animal survival.

There are several advantages to using small molecules, such as ISO-1 or ISO-66, to block MIF activity instead of neutralizing Abs against MIF or its receptor (9,12,16,41). Small-molecule inhibitors can be developed and synthesized via a less complex and expensive process, are non-immunogenic upon repetitive administration, are more mobile in reaching their target and do not usually require intravenous injection after formulation optimization [(12,42), and the present report]. Moreover, although administered at low concentrations, they can still effectively and specifically target MIF irrespective of its origin, i.e., whether deriving from host and/or cancer cells. Indeed, in our study 9 mg/kg of ISO-66 was shown to be non-toxic even after frequent administration and sufficient to enhance *in vivo* tumor-reactive immune responses leading to the reduction of tumor-cell expansion. In support of our last assumption, Yaddanapudi *et al* (5) most recently reported that the suicide inhibitor of MIF, 4-IPP, significantly slowed the rate of B16 tumor growth, but to intracellularly block MIF secretion by TAMs, 4-IPP was given at doses 10-fold higher (80 mg/kg) than ISO-66 in our models, raising specificity (35) and toxicity issues, if such high concentrations were to be adapted for clinical use. In another study, the drug ibudilast has been used in humans for two decades in the Far East for bronchial asthma and post-stroke complication. It is an allosteric inhibitor of MIF at clinically relevant concentrations, but its antitumor effect has yet to be studied (44).

Taken as a whole, our results in conjunction with accumulated evidence from other cancer studies, suggest that selective MIF inactivation upon treatment with ISO-66 restores the lytic ability of tumor-reactive CTLs and NK cells, which become immunocompetent. These cells can further efficiently kill tumor cells, thereby reducing the size of the tumors. Therefore, anti-MIF therapies using improved small molecule inhibitors selectively targeting MIF, such as ISO-66, may provide new treatment options with the potential to complement currently applied anticancer strategies.

## Figures and Tables

**Table I:** Crystallographic statistics.

Integration and scaling	
Space group	P3_1_21
Unit cell	a = b = 95.46 Å, c = 104.55 Å, α = β = 90°, γ = 120°
Resolution, Å (highest shell)	1.55 (1.61–1.55)
Unique reflections	79,934 (7,885)
Completeness, %	99.6 (99.2)
Redundancy	5.8 (5.3)
Avg. I/Avg. σ	33.2 (2.25)
R_merge_	0.058 (0.534)

Refinement	

R (working)	19.9%
R-free	22.0%
RMS deviations from ideality^a^	
Bond lengths (Å)	0.010
Bond angles (°)	1.273

Average B-factors^b^, Å^2^	

Overall	25.577
Protein (number of residues)	24.061 (114×3 chains)
Water	35.923 (281)
Chloride	34.078 (6)
Sulfate	28.648 (1)
Inhibitors	39.299 (4)

a Calculated by Refmac5, using reference values from Engh and Huber (43).

b Calculated using the Baverage program in the CCP4 suite (CCP41994).
